# The effect of lattice disorder on the low-temperature heat capacity of (U_1−*y*_Th_*y*_)O_2_ and ^238^Pu-doped UO_2_

**DOI:** 10.1038/s41598-019-51476-3

**Published:** 2019-10-21

**Authors:** Sorin-Octavian Vălu, Emanuele De Bona, Karin Popa, Jean-Christophe Griveau, Eric Colineau, Rudy J. M. Konings

**Affiliations:** 1 European Commission, Joint Research Centre (JRC), P.O. Box 2340, 76125 Karlsruhe, Germany; 2 Delft University of Technology, Faculty of Applied Sciences, Mekelweg 15, 2629 JB Delft, The Netherlands; 3 Laboratoire Structures, Propriétés et Modélisation des Solides, CNRS, CentraleSupélec, Université Paris-Saclay, 91190 Gif-sur-Yvette, France

**Keywords:** Nuclear fuel, Experimental nuclear physics, Phase transitions and critical phenomena

## Abstract

The low-temperature heat capacity of (U_1−*y*_Th_*y*_)O_2_ and ^238^Pu-doped UO_2_ samples were determined using hybrid adiabatic relaxation calorimetry. Results of the investigated systems revealed the presence of the magnetic transition specific for UO_2_ in all three intermediate compositions of the uranium-thorium dioxide (*y* = 0.05, 0.09 and 0.12) and in the ^238^Pu-doped UO_2_ around 25 K. The magnetic behaviour of UO_2_ exposed to the high alpha dose from the ^238^Pu isotope was studied over time and it was found that 1.6% ^238^Pu affects the magnetic transition substantially, even after short period of time after annealing. In both systems the antiferromagnetic transition changes intensity, shape and Néel temperature with increasing Th-content and radiation dose, respectively, related to the increasing disorder on the crystal lattice resulting from substitution and defect creation.

## Introduction

The regular substitution of one or more atoms in the crystal lattice of solid solution phases can affect their thermodynamic properties significantly. Since a substitutional atom has different characteristics compared to the original one, substitution can lead to lattice adjustment and strain, lattice disorder, breaking of magnetic order, etc., which all can affect the phonon states and their dispersion in the material and thus the thermal capacity and the thermal transport. Effects on the thermal capacity are generally observed at low temperature, well below the limit of 3*R* per mole as established by Einstein and Debye, and can be studied with low-temperature calorimetric techniques.

All tetravalent actinide ions can be hosted in the AnO_2_ dioxide close-packed fluorite structure and complete solid solution series exists between all end members. Low-temperature calorimetric measurements of (Th_1−*y*_Pu_*y*_)O_2_^1^ showed that there is a substantial composition effect and the excess heat capacity was explained by the strain resulting from the substitution of isovalent ions of significantly different size on the cation sublattice, 96 pm for Pu^4+^ and 105 pm for Th^4+^. Similar measurements on (U_1−*y*_Am_*y*_)O_2−*x*_ with *y* = 0.08 and 0.20 yielded less obvious results^2^. Again a substantial excess heat capacity was observed at very low temperature but also the complete absence of the magnetic transition due to the antiferromagnetic ordering of the U^4+^ ions even at a minor extent of dilution. However, the (U_1−*y*_Am_*y*_)O_2−*x*_ solid solution is complicated by the fact that a charge transfer takes place and that americium becomes trivalent, compensated by a equimolar amount of pentavalent uranium. This results in a more complex (U^4+^,U^5+^,Am^3+^)O_2_ composition, and thus a higher “dilution” effect than formally. Moreover, americium is a highly radioactive material (half life 432.2 years) and radiation effects due to alpha decay of ^241^Am create disorder that also may affect the phonon states and dynamics.

In order to obtain information on the separate effects of dilution-induced disorder and radiation-induced disorder on the heat capacity of actinide oxides and particularly on the magnetic transition in uranium-based fluorite dioxide systems we have performed studies on two highly different materials. Firstly, we measured the low-temperature heat capacity of (U_1−*y*_Th_*y*_)O_2_ with *y* = 0.05, 0.09 and 0.12. In this material the non-magnetic Th^4+^ acts as diluent for the magnetic U^4+^ ions (no charge transfer), whereas the additional effect of distortion in the lattice due to the mismatch of the ionic radii (103 pm for U^4+^ and 108 pm for Th^4+^) is expected to be relatively small. Both elements are only mildly radioactive, and their handling allows to obtain heat capacity data of the highest quality and accuracy with the instrument employed, and radiation-induced disorder is negligible. Secondly, we measured the low-temperature heat capacity of uranium dioxide doped with the highly radioactive ^238^Pu isotope (half life 87.7 years) as a function of aging time to assess the effect of alpha decay and the concomitant lattice disorder in the fluorite structure.

## Results and Discussion

### (U_1−*y*_Th_*y*_)O_2_: effect of matrix dilution and lattice strain

**Figure 1:**
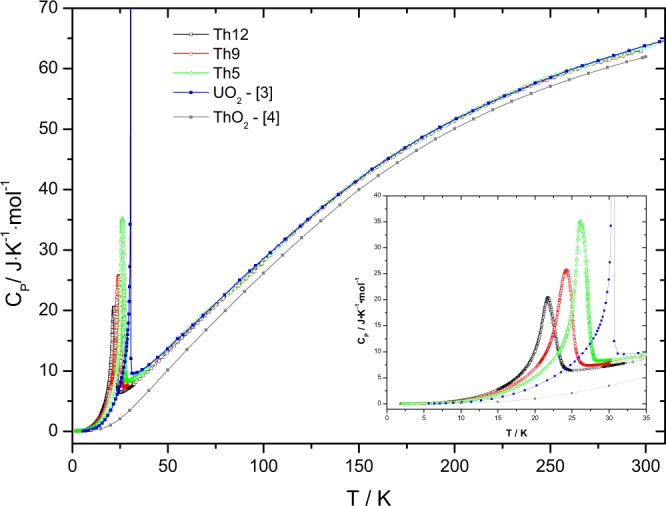
The low-temperature heat capacity of the Th5, Th9 and Th12 samples studied in this work.

The heat capacity of (U_1−*y*_,Th_*y*_)O_2_ solid solution with y = 0.05, 0.09 and 0.12 is shown in Fig. 1 together with the heat capacity of UO_2_^3^ and ThO_2_^4,5^. *λ*-type anomalies are observed around 27.2 K, 25.1 K and 21.8 K, respectively for the intermediate compositions Th5, Th9 and Th12 with a maximum C_*P*_ (J · K^−1^ · mol^−1^) of 33.3, 23.3 and 20.5, respectively. This heat capacity anomaly is related to the long range antiferromagnetic ordering of the spins of the U^4+^ ions in UO_2_ at low temperature, which was first observed by Jones *et al*.^6^ and analysed in detail later by Huntzicker and Westrum^3^. The nature of the transition has been further analyzed by neutron diffraction^7,8^ and NMR studies^9^. These studies showed that at low temperature the oxygen cage of the UO_2_ unit cell shows a Jahn-Teller distortion. The physics of the transition is very complex and is still subject of investigations. It is beyond the scope of this work and for details the readers are referred to the review by Lander and Caciuffo^10^.

This peak amplitude could be due to the fact that Huntziker and Westrum made a very detailed scan through the transition region (temperature step < 0.01 K) whereas we made a standard scan (>0.1 K). On the other hand it is a known weak point of our technique that it cannot accurately fit the time dependent relaxation data in case of a large heat capacity dependence near T_*N*_, as in the case of UO_2_. This should be taken into account when discussing the entropy values.

**Figure 2:**
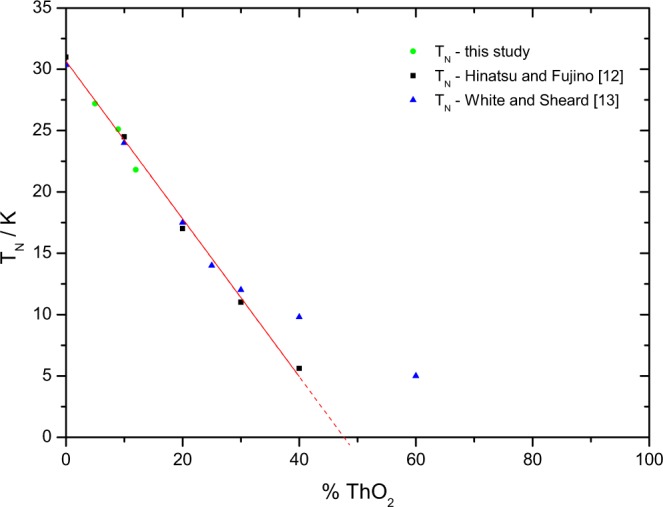
The Néel temperature of the (U,Th)O_2_ solid solution as function of composition.

The current results clearly show that with increasing thorium concentration in the solid solution the antiferromagnetic peak shifts towards lower temperature, its intensity is reduced and area broadens. This is consistent with earlier studies of the (U,Th)O_2_ solid solution. Both Comly^11^ and Hinatsu and Fujino^12^ studied the magnetic transition by magnetic susceptibility measurements and found a linear dependence of T_*N*_ (see Fig. 2). White and Sheard^13^ found that T_*N*_ derived from thermal expansion decreases linear up to 30% ThO_2_ and extrapolates to a similar value. However, the experimental data suggest that above 30% ThO_2_ the slope of dependence changes.

**Figure 3:**
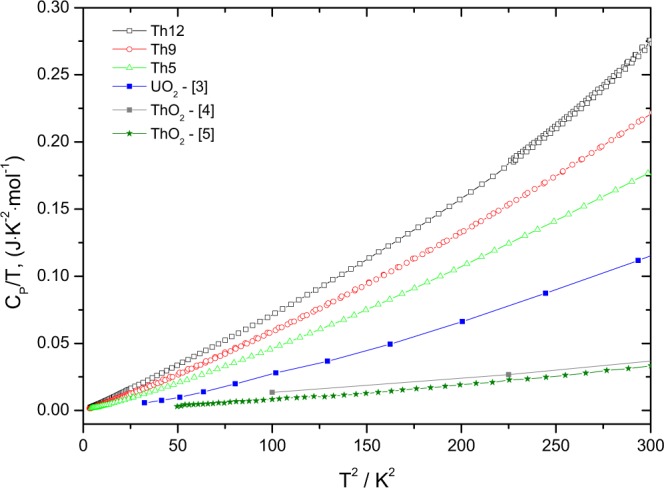
Low-temperature heat capacity of the Th5, Th9 and Th12 samples, together with the heat capacity data of UO_2_ obtained by Huntzicker and Westrum^3^, ThO_2_ of Osborne *et al*.^4^ and Magnani *et al*.^5^.

When plotting *C*_*P*_/*T* over the squared temperature, as shown in Fig. 3, it can be observed that the heat capacity reaches zero at 0 K absolute temperature, which is the situation of a perfect crystal implied by the third law of thermodynamics. For all compositions the nearly linear curve is in good agreement with the Debye theory. However, the low-temperature values for the (U_1−*y*_Th_*y*_)O_2_ solid solutions are well above those of the end members, UO_2_ and ThO_2_, revealing the significant excess heat capacity.

**Figure 4:**
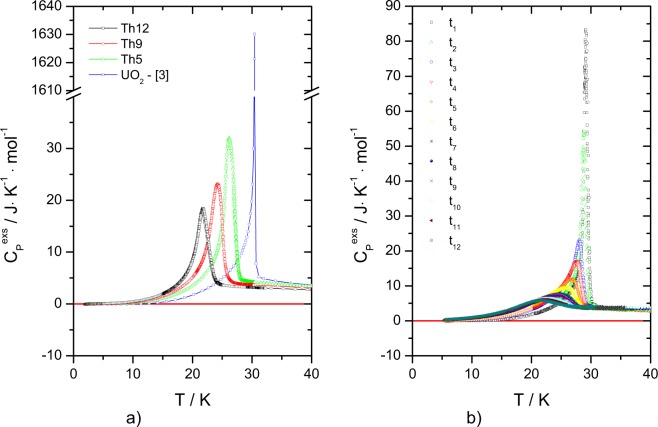
The low-temperature magnetic transition observed in this work plotted as ${C}_{P}^{exs}$ for a) (U_1−*y*_,Th_*y*_)O_2_ solid solution (Th5, Th9, Th12 and literature data of UO_2_^3^) and b) (U,^238^Pu)O_2_ as a function of storage time.

For a further analysis of the magnetic anomalies, the “ideal” heat capacity (Neumann-Kopp) of the mixed oxides was estimated by interpolation based on the low-temperature literature data of UO_2_^3^ and ThO_2_^4^ end-members, and by neglecting the magnetic transition for UO_2_ using the lattice heat capacity suggested by Huntzicker and Westrum^3^. The experimental results of (U_1−*y*_Th_*y*_)O_2_ solid solution are in a good agreement with the computed values from the Neumann-Kopp rule and no significant difference for the measured temperature interval is observed, except the low-temperature range below the characteristic antiferromagnetic transition. Further, we have calculated the difference between the two heat capacity curves (measured and computed) and will refer to it as the excess heat capacity (${C}_{P}^{exs}$), which is shown in Fig. 4 and defined as:

1 $${C}_{P}^{exs}={C}_{P}^{m}-{C}_{P}^{NK}$$

where ${C}_{P}^{m}$ is the measured heat capacity and ${C}_{P}^{NK}$ is the computed heat capacity by Neumann-Kopp’s rule disregarding the anomaly of the UO_2_. This excess heat capacity primarily reflects the effect of magnetic fluctuation of the metal sublattice substitution on the phonon dynamics. Moreover, a weak Jahn-Teller distortion or CF transitions play a role in UO_2_ up to temperatures well beyond 200 K^14^, but it is not obvious whether this effect is influenced by dilution in the (U,Th)O_2_ solid solution. Overall, the N-K approximation is providing a straightforward approach to assess this and thus the ${C}_{P}^{exs}$ will include all these effects.

Each individual peak has been been integrated using Origin Software (version 8.1) and the results are shown in Table 1 and compared to similar analysis done for literature data of UO_2_. The expected entropy of the triplet ${\Gamma }_{5}$ ground state of the ^3^H_4_ multiplet of U^4+^ in pure UO_2_ is *R* ln(3) = 9.1 J · K^−1^ · mol^−1^. Huntzicker and Westrum^3^ reported 8.4 J · K^−1^ · mol^−1^ for the total anomaly and ~6.3 J · K^−1^ · mol^−1^ at the Néel temperature. The latter becomes 7.24 J · K^−1^ · mol^−1^ using the same analysis as for our measurements.

**Table 1:** Data of peak analysis of (U_1−*y*_,Th_*y*_)O_2_ solid solution with y = 0.05, 0.09 and 0.12 together with the one of UO_2_^3^.

Sample	T_*peak*_ (K)	S_*AF*_ (J · K^−1^ · mol^−1^)	Peak height (J · K^−1^ · mol^−1^)
UO_2_	30.44	7.24	1635.9
Th5	27.2	4.64	33.3
Th9	25.1	4.26	23.3
Th12	21.8	3.74	20.5

The entropy values associated to the magnetic transition of (U_0.95_Th_0.05_)O_2_, (U_0.91_Th_0.09_)O_2_ and (U_0.88_Th_0.12_)O_2_ at the Néel temperature were found to be 4.64, 4.26 and 3.74 J · K^−1^ · mol^−1^, respectively (corresponding to 4.7, 4.7 and 4.3 J · K^−1^ · mol(U)^−1^). These values should not be compared to the values for pure UO_2_ by Huntziker and Westrum because of the different calorimetric techniques. As stated earlier, Huntziker and Westrum made a very detailed scan through the transition region (temperature step < 0.01 K) resulting in a very sharp peakw, which our measurement technique does not capture fully. It is also a known weak point of the PPMS technique that it cannot accurately fit the time dependent relaxation data in case of a large heat capacity dependence near TN^15^, as in the case of UO_2_.

Linear extrapolation of the values for the entropy of transition derived from our measurements indicates that the anomaly disappears at about 44% of thorium in the mixed solid solution, which means when five of the twelve second-nearest U neighbours in the UO_2_ fluorite lattice are substituted by Th, breaking the long-range magnetic order of U^4+^. This is close to the value 42% found by Comly^11^ and about 45% Th by Hinatsu and Fujino^12^ by magnetic susceptibility measurements.

### (U,^238^Pu)O_2_: effect of radiation damage

**Figure 5:**
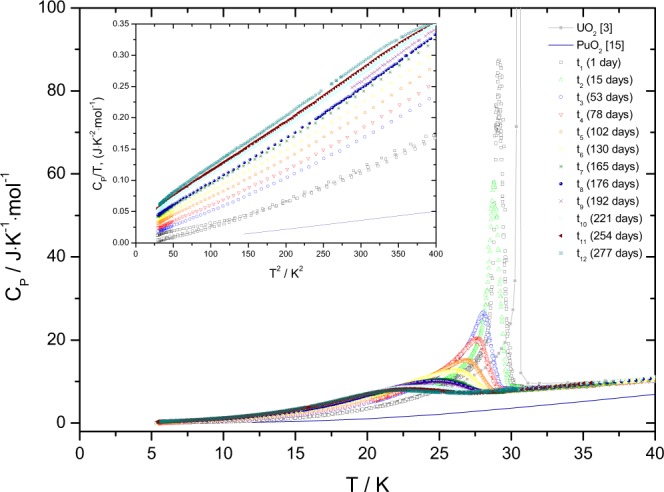
The low-temperature heat capacity of the (U,^238^Pu)O_2_ sample studied in this work together with PuO_2_ obtained by Flotow *et al*.^26^ and UO_2_ previously measured in house.

The results of the series low-temperature heat capacity measurements of the (U,^238^Pu)O_2_ sample in the temperature interval from 5 to 50 K for different storage times after annealing of the sample are presented in Fig. 5, together with the low-temperature heat capacity data of the end-members. It is evident that the *λ* transition rapidly changes shape and position with the aging time, c.q. accumulated dose.

**Figure 6:**
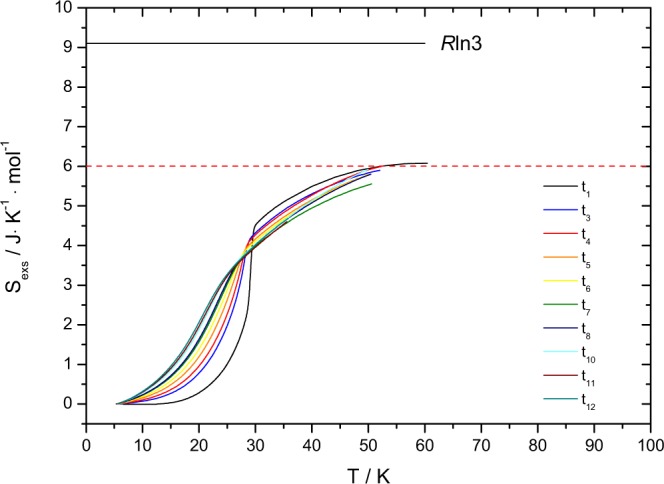
Excess entropy of ^238^Pu-doped UO_2_ sample. Different coloured curves of S_*exs*_ indicate the specific time (and accumulated radiation damage) at which the measurements took place. Some of the curves are not shown because their measurements have not been extended to the considered temperature interval.

The excess heat capacity peak of the measured sample has been derived as a function of the accumulated *α* dose up to 277 days. Using again the Origin Software (version 8.1), the peak evaluation has been performed and the related thermodynamic data are given in Table 2. Applying the estimated heat capacity of UO_2_ based on the same corrections made by Huntzicker and Westrum^3^ the excess entropies at the Néel temperature of the magnetic transition are found to decrease with time (Table 2). Integrating over the full temperature range to T = 50 K, we found an excess entropy of about 6 J · K^−1^ · mol^−1^ for all measurements as shown in Fig. 6, which means that even if the anomaly vanishes the corresponding entropy per mole is largely distributed over much wider temperature interval. The inset graph from Fig. 5 shows that when *C*_*P*_/*T* is plotted over the squared temperature, the heat capacity data does not become zero at 0 K, which indicates the presence of lattice disorder and residual entropy typical for a non-equilibrium state.

**Table 2:** Peak analysis data of (U,^238^Pu)O_2_ solid solution obtained on different stages of radiation damage accumulation.

Notation	Center (K)	S_*AF*_ (J · K^−1^ · mol^−1^)	Peak height (J · K^−1^ · mol^−1^)
1 day	29.3	4.58	87.3
15 days	28.8	−^*a*^	58.0
53 days	28.1	4.25	26.7
78 days	27.6	4.19	20.5
102 days	26.9	4.04	15.2
130 days	26.1	3.92	12.5
165 days	25.2	3.80	10.5
176 days	24.9	3.77	10.2
192 days	24.9	3.65	9.9
221 days	23.4	3.61	8.8
254 days	22.7	3.38	8.2
277 days	22.3	3.28	7.9

^*a*^The excess entropy at t_2_, corresponding for the measurement after 15 days, could not be calculated due to the minimal temperature interval of the considered measurement.

**Figure 7:**
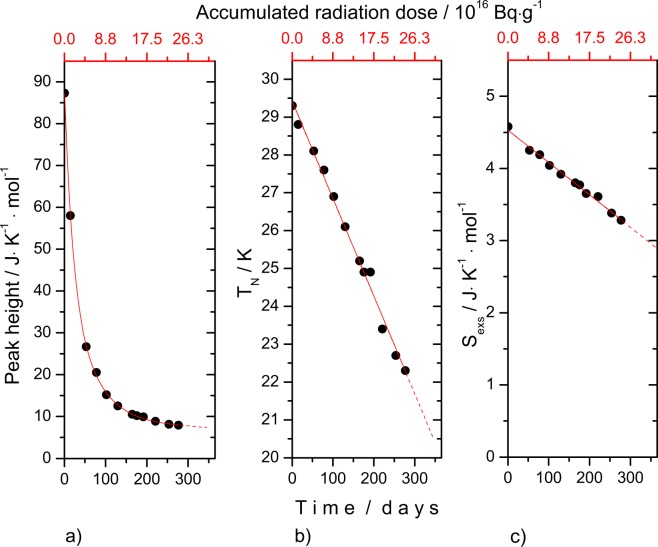
Thermodynamic characteristics of the low-temperature heat capacity antiferromagnetic anomaly of the (U,^238^Pu)O_2_ sample studied in this work. (**a**) The height of the anomaly at the maximum; (**b**) the Néel temperature and (**c**) the entropy of transition at the Néel temperature.

As shown in Fig. 7a the peak height of the magnetic transition decreases significantly with a accumulated dose (time) and the area broadens, similar to the composition dependence observed for the (U_1−*y*_Th_*y*_)O_2_ solid solution. Moreover, the maximum of the peak (shown as Néel temperature in Fig. 7b) is shifting towards lower temperatures and the corresponding entropy at the Néel temperature decreases linearly as well (Fig. 7c). Note that we do not have a value for t_0_ because the heat capacity of damage-free (U,Pu)O_2_ with about 2% Pu_*total*_, used in this work, has not been measured. Pure UO_2_ cannot serve as reference, as our results for (U_1−*y*_,Th_*y*_)O_2_ show that the heat capacity is already affected at low concentrations.

Our results show that the antiferromagnetic ordering of the spins of the U^4+^ ions in uranium dioxide is dramatically affected by the continuous self-irradiation from the high *α* activity of ^238^Pu, which leads to atomic displacements in the lattice and the subsequent formation of point defects and extended defects. Each *α*-decay produces about 1500–2000 displacements, which accumulate over time in substantial lattice disorder. In about 24 hours after annealing the intensity of the heat capacity peak specific to UO_2_ has diminished to about 5% from its original intensity, and further flattening of the peak occurs with increasing alpha-decays per mole of material. Since the change in the entropy is minimal, it must be concluded that the antiferromagnetic transition gradually changes into a Schottky-type anomaly with a significant part of the residual entropy redistributed over the entire temperature interval. This can be understood from the changes in the phonon dynamics and anharmonicity due to radiation induced disorder created by the strong ^238^Pu alpha emitter and magnetoelastic interaction between the magnetic moments in the uranium atoms and lattice distortions.

## Conclusions

**Figure 8:**
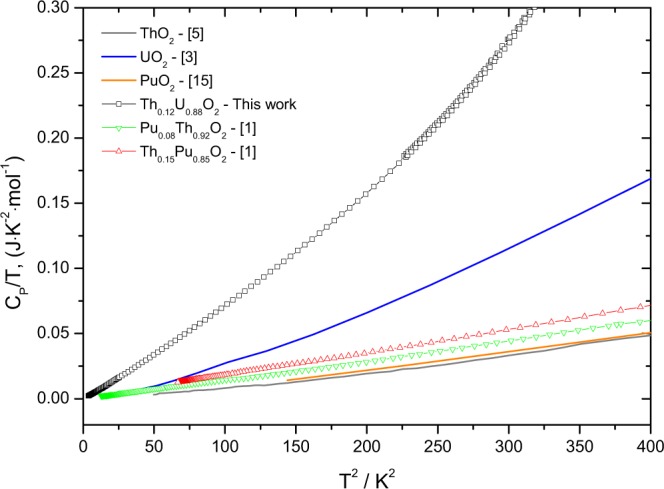
The low-temperature heat capacity of the Th_0.12_Pu_0.88_O_2_ (Th12) sample studied in this work, together with the end-members UO_2_, ThO_2_ and PuO_2_ and Th_1−*y*_Pu_*y*_O_2_ (y = 0.08 and 0.85) plotted as Cp/T vs T^2^.

The heat capacity of a fluorite-structured mixed dioxide is strongly driven by the interaction of the phonons with the metal atoms of the end-members of the solid solution. Taking into account that both uranium and thorium have long half lives, lattice disorder (displacements and vacancies) resulting from *α* self-irradiation in (U_1−*y*_Th_*y*_)O_2_ is limited in this system, and the principal disorder mechanism is substitution on the metal sublattice. Our analysis of the antiferromagnetic transition in (U_1−*y*_Th_*y*_)O_2_ shows that the substitution of five of the twelve uranium atoms in the unit cell by thorium suppresses the long-range magnetic ordering in the solid solution. The cation size difference between U^4+^ (100 pm) and Th^4+^ (105 pm) is smaller than between Pu^4+^ (96 pm) and Th^4+^, but the excess heat capacity at low temperature (<20 K) is substantially larger than for a similar extent of substitution in (Th_1−*y*_Pu_*y*_)O_2_ samples measured previously^1^, as shown in Fig. 8 the plot of C_*P*_/T vs T^2^. We attribute this to the broadening of the *λ*-transition and its shift and extension to lower temperatures with increasing extent of substitution; the excess heat capacity due to lattice strain being masked in the tail of the transition, which changes from a sharp *λ*-type peak to a more broad and diffuse transition. This phenomenon is known from other antiferromagnetic materials such as Zn_2_VO(PO_4_)_2_ doped with Ti^4+^^16^, doped LaSr_2_Mn_2_O_7_^17^, or Mg(Al,Cr)_2_O_4_^18^.

The heat capacity of both (U_1−*y*_Th_*y*_)O_2_ and (Th_1−*y*_Pu_*y*_)O_2_ extrapolate to zero at 0 K, indicating perfect crystal properties and no effect of substitution on the metal sublattice. In contrast the heat capacity of (U,^238^Pu)O_2_ sample shows a significant residual heat capacity at 0 K which can be attributed to disorder caused by self-radiation, since the residual value clearly increases with time/dose. We conclude that the principal causes for this are the creation of uranium Frenkel pairs and oxygen Frenkel pairs in the crystal lattice, and extended defects at high doses. With increasing dose (c.q. concentration of defects) the antiferromagnetic transition in this sample transforms from a sharp peak to a broad anomaly. According to X-ray diffraction measurements, the lattice expansion due to accumulated dose (time) reaches saturation (E. De Bona, personal communication) in this sample after about 300 days and results in a disordered quasi-equilibrium state in which defect creation and recombination balance each other. Staicu *et al*.^19^ concluded from thermal annealing experiments of ^238^Pu-doped UO_2_ that both oxygen and uranium interstitials are present in the material. Moreover, defects that escape from the recombination can concentrate into loops and voids^20^. Molecular dynamics simulations of alpha decay in UO_2_ by Van Brutzel and Rarivomanantsoa^21^ showed that the number of oxygen Frenkel pairs is twice as high as the uranium Frenkel pairs, and that the number of displaced atoms in stable point defects at the end of the cascade is 13% for uranium and about 2% for oxygen. Thus it can be concluded that the disordered quasi-equilibrium state has not yet been achieved in our experiments after 277 days and further change in the transition thermodynamics can be expected.

It is interesting to hypothesise how the radiation affects the electronic states of U^4+^ in the damaged material. The crystal field splitting of the U^4+^ ground state multiplet in paramagnetic UO_2_ results in a ${\Gamma }_{5}$ triplet ground state, with a ${\Gamma }_{3}$ doublet as first excited state at 150 meV (Magnani *et al*.^22^). Considering that both Uranium Frenkel Pairs (UFP) and Oxygen Frenkel Pairs (OFP) are formed in the damaged crystal lattice, the Néel transition in the (U,^238^Pu)O_2_ sample will no longer correspond to a disorder-order transformation, but to a transition between two disordered states with reduced long-range magnetic ordering. The strong lattice disorder in the material with formation of UFPs and OFPs will lead to changes in the dynamics between the U^4+^ and the surrounding O neighbours and likely results in a splitting of the ${\Gamma }_{5}$ ground state leading to two low-lying electronic states in the disordered material. As result the sharp transition changes to a Schottky-like anomaly.

**Figure 9:**
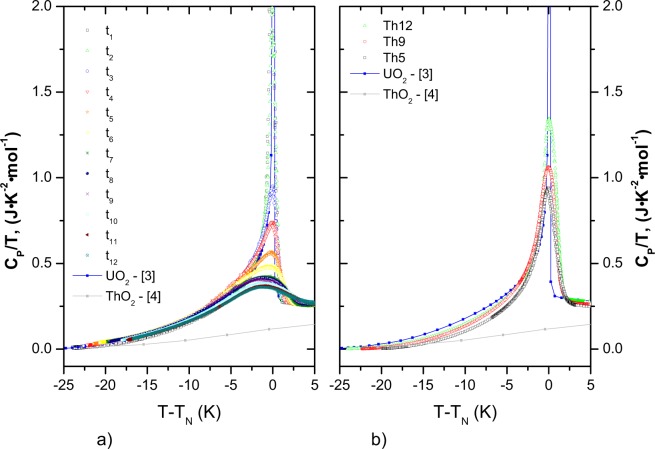
The low-temperature magnetic transition aligned at peak center as Cp/T vs T-T_*N*_ for (**a**) (U,^238^Pu)O_2_ as a function of time and (**b**) (U_1−*y*_,Th_*y*_)O_2_ solid solution (Th5, Th9, Th12 and literature data of UO_2_^3^).

In Fig. 9 we aligned the results for the magnetic transition in both systems at the peak center by using *T - T*_*N*_ as temperature axis (the reason for negative values). Although, the overall change in peak shape of the two investigated systems is comparable, the strong asymmetry of the ^238^Pu-doped system evidences that disorder on the metal and oxygen sublattice may lead to similar magnetoelastic effects.

In conclusion we can state that the antiferromagnetic heat capacity anomaly in UO_2_ is highly sensitive to disorder in the crystal lattice, as discussed by Huntzicker and Westrum^3^ in their seminal paper. They explained the difference between their results and those by Jones *et al*.^6^, that revealed a much less pronounced anomaly at a lower temperature, by the presence of oxygen interstitials as a result of a hyperstoichiometric composition of the UO_2.01_ sample. We, here, have shown that lattice disorder caused by metal ion dilution and radiation damage has a similar effect on the transition characteristics, with a measurable impact at already low concentrations and moderate doses.

## Material and Methods

### Sample preparation and characterisation

(U,Th)O_2_ samples with three Th concentrations (5, 9 and 12 mol %) were prepared using a hydrothermal method^23,24^. The synthesis was conducted under argon and in the presence of minute amounts of hydrazine in order to limit the oxidation of the U^4+^. As starting materials we have used solutions of Th^4+^ (1.9 M in 8 M HNO_3_) and U^4+^ (0.47 M, obtained by electroreduction of UO_2_(NO_2_)_2_ solution in 4 M HNO_3_ containing 0.5 M of hydrazine). The other reagents (NH_4_OH 25%, hydrazine hydrate) were of analytical grade and used as supplied by Merck.

The hydroxides were firstly produced by direct coprecipitation of the An^4+^ solutions in nitric acid with ammonium hydroxide (10–20% excess). The obtained coprecipitates were washed several times with distilled water in order to remove any trace of nitrate, which may induces the oxidation of U^4+^ to soluble U^6+^ under the working temperature conditions. The decomposition was conducted under hot compressed water for 5 h at 250 °C in teflon autoclaves. The resulting nanocrystals (circa 3.5 nm) were washed with water, ethanol and acetone, in order to gradually decrease the polarity of the solution. Pellets of 3.5 mm diameter and several mm in height were produced by pressing and heated for 5 h at 1000 °C (1^*st*^ step) and 24 h at 1650 °C (2^*nd*^ step) under Ar-H_2_ (4%).

The real U and Th concentration in the samples was measured by Inductively Coupled Plasma Mass Spectrometry (ICP-MS) using a A ThermoFinnigan Element instrument with auto-sampler, according to an appropriate standardisation method. The results are summarized in Table 3.

**Table 3:** Notation, composition and fabrication data of the studied samples.

Notation	Composition^*a*^ Th/(U + Th) (mol%)	Heat treatment Ar/H_2_	Sintering Ar/H_2_	Cell parameter (Å)
Th5	4.78 ± 0.02	1000 °C, 5 h	1650 °C, 24 h	5.4745 (1)
Th9	8.76 ± 0.04	1000 °C, 5 h	1650 °C, 24 h	5.4800 (1)
Th12	12.30 ± 0.05	1000 °C, 5 h	1650 °C, 24 h	5.4853 (2)
Notation	Composition ^*a*238^ Pu (%wt)	Calcination (Ar/H _2_ )	Sintering (Ar/H _2_ )	Cell parameter (Å)
(U,^238^Pu)O_2_	1.6	700 °C, 2 h	1650 °C, 6 h	5.4688

^*a*^Composition measured ICPMS, k = 2.

The UO_2_-^238^PuO_2_ sample was obtained by pressing and sintering of a powder produced via liquid route, guaranteeing a homogeneous and intimate mixing. The metal content of the ^238^PuO_2_ was 87%wt Pu, 73%wt being ^238^Pu, the remaining 13% being the decay product uranium 234. The composition was chosen considering the activity of the dopant powder and tuning the dopant concentration to fit the aging of the sample to a reasonable period of time. The Pu dopant powder was dissolved as received using HNO_3_ and dropwise addition of HF, and then added to the corresponding amount of uranyl solution. Precipitation was achieved by adding ammonia to the solution, and the resulting precipitate was washed with distilled water several times and then filtered on paper filters. The paper filters were burnt away during a pre-calcination step (6 h at 400 °C) under air and then the powder was calcined at 700 °C for first 2 h in air (N_2_ + 20%O_2_) to remove any remaining paper and finally for 2 h under Ar-H_2_ (4%). This powder was then pressed in 5 mm disks of roughly 80 mg each with a force of 14.5 kN (738 MPa) and sintered for 6 h under Ar-H_2_ at 1650 °C. Based on elemental and isotopic composition analysis done in March 2016 by ICPMS the actual composition of the investigated sample is (^234^U_0.0037_^235^U_0.0070_^238^U_0.9680_^238^Pu_0.0156_^239^Pu_0.0047_^240^Pu_0.0008_)O_2_ and represents 99.98%. The diference of 0.02% is given by the sum of the isotopes ^241^Am, ^233^U and ^236^U. From chemical point of view the sample contains just uranium and plutonium being virtually free of americium and other impurities. We will refer to this composition as (U,^238^Pu)O_2_. Table 3 lists the fabrication data.

### X-ray analysis

**Figure 10:**
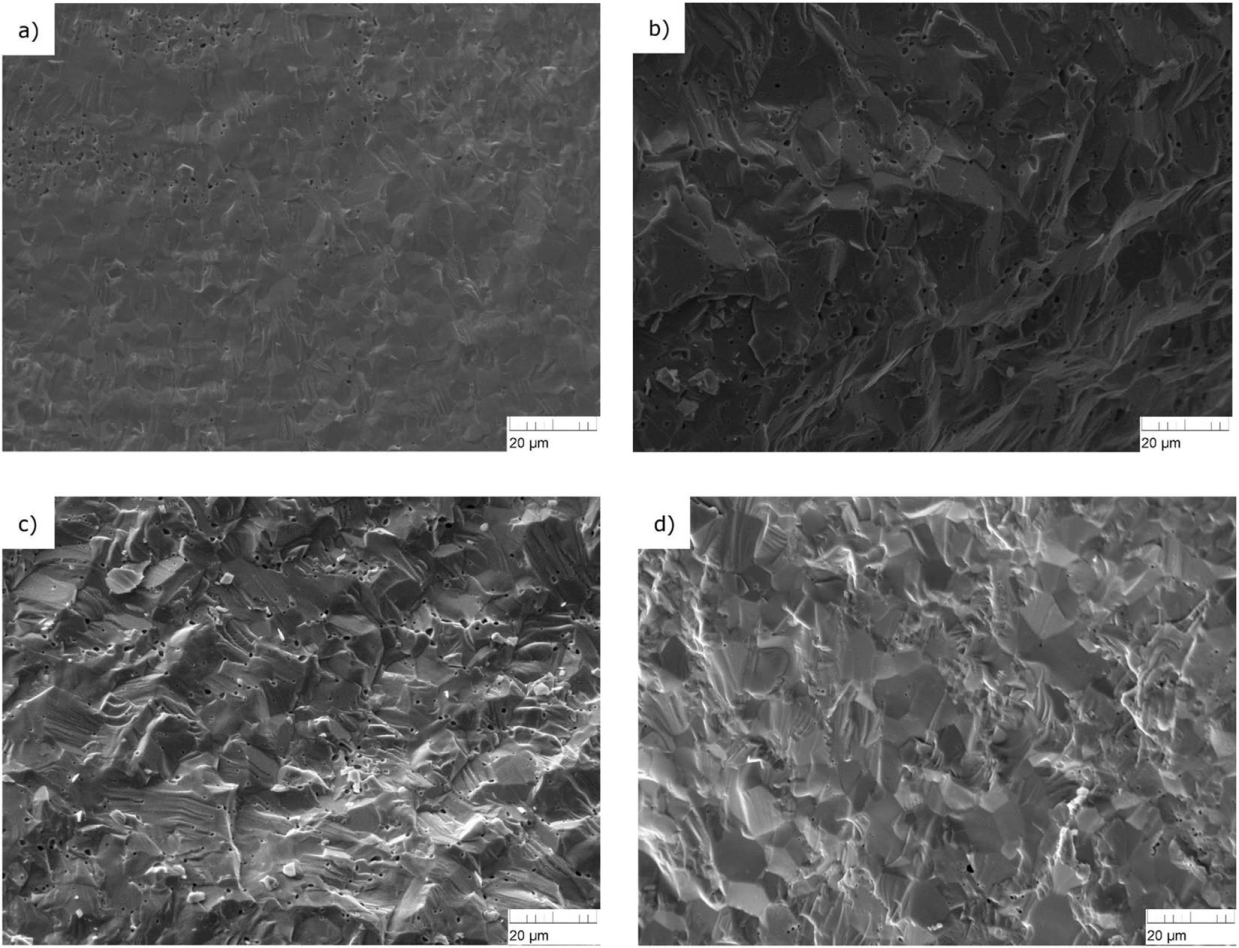
Scanning electron microscopic (SEM) images of the (U_1−*y*_Th_*y*_)O_2_ samples: (**a**) Th5, (**b**) Th9 and (**c**) Th12 and (U,^238^Pu)O_2_ sample in (**d**).

During and after the production, samples were characterized by XRD using a Bruker D8 (Bruker AXS GmbH, Karlsruhe, Germany) diffractometer mounted in a Bragg–Brentano configuration with a curved Ge (1,1,1) monochromator and a ceramic copper tube (40 kV, 40 mA) and supplied with a LinxEye position sensitive detector. The cell parameters of the investigated compositions derived from a Rietveld analysis, are listed in Table 3. The resulting values for the (U,Th)O_2_ samples agree excellently with the cell parameters derived from Vegard’s law. Also the value for the (U,^238^Pu)O_2_ sample corresponds well to the lattice parameter derived Vegard’s law considering the actual composition. This confirms that all our samples are homogeneous solid solutions of stoichiometric composition. Homogeneity of the mixtures is evident also in the SEM images (Fig. 10) that have been taken after production.

### Calorimetry

The heat capacity of (U_1−*y*_Th_*y*_)O_2_ with y = 0.05, 0.09 and 0.12 was measured using a low-temperature vacuum calorimeter based on a hybrid adiabatic relaxation method (PPMS, Quantum Design Inc.) as described in our previous paper^2^. The measurements of the Th5, Th9 and Th12 samples were carried out using small solid pieces with the masses of 47.4 mg, 37.1 mg and 57.1 mg, respectively, in the temperature intervals of 2.05 to 304.10 K, 1.86 to 292.93 K and from 1.94 to 297.50 K. Different from our measurements of other actinide oxide solid solutions^1,2^, these samples have been measured without protective encapsulation into Stycast (for radioprotection purposes) which results in a high accuracy of the PPMS apparatus with estimated uncertainties for the heat capacities of about 1 to 2% as reported by Lashley *et al*.^15^.

In the case of (U,^238^Pu)O_2_ sample, the same instrument has been used for measuring the heat capacity as a function of aging time. Since the main objective of this study was to study the behavior of the magnetic peak, we have focused our investigation in the interested temperature region and measurements have been performed in the temperature interval from about 5 K to 50 K. The sample consisted of one small solid piece with mass of 1.4 mg and for radiation protection reason it was wrapped in Stycast^25^ which increased the uncertainty to about 4 to 5% after the corresponding heat capacity was subtracted. Before the first measurement, the sample was annealed for 4 hours into Ar/H_2_ atmosphere (1 L/min.) at 1650 °C to eliminate the self-radiation damage in the lattice accumulated during the storage at room temperature in the period between fabrication and measurement. After annealing and Stycast encapsulation the first measurements have been carried out after about 24 hours and we consider this moment as being the reference time point (t_1_) with minimum radiation damage. The subsequent measurements have been performed after different time intervals, 15 days (t_2_), 53 days (t_3_), 78 days (t_4_), 102 days (t_5_), 130 days (t_6_), 165 days (t_7_), 176 days (t_8_), 192 days (t_9_), 221 days (t_10_), 254 days (t_11_) and 277 days (t_12_) for better understanding the influence of the cumulative radiation dose on the thermal properties of the mixed oxide. In between the measurements the sample was stored at room temperature.

## Data Availability

The raw/processed data required to reproduce these findings cannot be shared at this time due to technical and time limitations.
